# The Cancer Menace

**Published:** 1917-02

**Authors:** 


### The Cancer Menace.

Authorities now seem pretty well agreed that cancer is neither hereditary nor contagious, but the statistics show that it is none the less to be considered as one of the diseases hardest to cope with and most to be dreaded. The death rate from cancer, according to the latest statistics, is 79.4 per 100,000, and shows an almost continuous increase from the time that the figures have been kept with much degree of accuracy. The hope for cure of the disease seems to rest largely in early treatment, and the great mortality comes from failure on the part of those who have it to secure treatment in its incipient stages. For some reason, cancer patients are either ignorant of the character of the disease until it gets into an advanced stage or else are reticent about communicating their knowledge to the family physician. It is an insidious disease, causing little pain at first, and most people do not call on a physician or surgeon until they suffer pain which they wish treated rather than whatever disease causes the pain.

The mortality from cancer can be greatly reduced through a systematic and thorough educational campaign. Unfortunately, the physician has little means of discovering it in his practice in its early stages, but the people should be taught to know the indications of the approach and existence of cancer, and should be impressed with the importance of early treatment. To do this the press and the schools must be used and physicians and surgeons who will take it on themselves to do this educational work will be benefactors to the communities where they disseminate the information. First rid the people of the dread of contagion and of inheriting the disease, then show the importance of watching for its appearance, and urge early efforts to stop its deadly growth.

There is more to the medical profession than merely treating the patients who come to the office for medical advice and treatment. The really worthy physician should realize the opportunities afforded by his professional knowledge for rendering needful public service, and should not through any false sense of modesty hesitate to perform his public duties. Sensible people will not regard health campaigns conducted by physicians as self-advertising, and a physician worthy of the name will not care what the other kind of people think or say about him.

